# Tunneling nanotubes, TNT, communicate glioblastoma with surrounding non-tumor astrocytes to adapt them to hypoxic and metabolic tumor conditions

**DOI:** 10.1038/s41598-021-93775-8

**Published:** 2021-07-15

**Authors:** Silvana Valdebenito, Shaily Malik, Ross Luu, Olivier Loudig, Megan Mitchell, George Okafo, Krishna Bhat, Brendan Prideaux, Eliseo A. Eugenin

**Affiliations:** 1 Department of Neuroscience, Cell Biology, and Anatomy, University of Texas Medical Branch (UTMB), Research Building 17, Fifth Floor, 105 11th Street, Galveston, TX 77555 USA; 2 Center for Discovery and Innovation, Hackensack Meridian Health, Nutley, NJ USA; 3 GO Pharma-Consulting Ltd., Welwyn, UK; 4 Department of Translational Molecular Pathology, Division of Pathology and Laboratory Medicine, M.D. Anderson, Houston, TX USA

**Keywords:** CNS cancer, Cancer microenvironment

## Abstract

Cell-to-cell communication is essential for the development and proper function of multicellular systems. We and others demonstrated that tunneling nanotubes (TNT) proliferate in several pathological conditions such as HIV, cancer, and neurodegenerative diseases. However, the nature, function, and contribution of TNT to cancer pathogenesis are poorly understood. Our analyses demonstrate that TNT structures are induced between glioblastoma (GBM) cells and surrounding non-tumor astrocytes to transfer tumor-derived mitochondria. The mitochondrial transfer mediated by TNT resulted in the adaptation of non-tumor astrocytes to tumor-like metabolism and hypoxia conditions. In conclusion, TNT are an efficient cell-to-cell communication system used by cancer cells to adapt the microenvironment to the invasive nature of the tumor.

## Introduction

Tunneling nanotubes (TNT), also called cytonemes in Drosophila and tumor microtubes in mouse tumors, are the only cell-to-cell communication system that enables the long-range exchange of cytoplasmic factors and organelles between connected cells. TNT are cellular processes that enable cell-to-cell communication at distances from 30 to 500 µm. Several groups have suggested that TNT, cytonemes, and tumor microtubes are different structures, but without specific markers or detailed characteristics, these claims are unsupported and require further research^1^. Normally, TNT formation and associated intercellular communication participate in key biological processes, including development, signaling, and the immune response, but TNT formation during healthy conditions is minimal^1–8^. We and others demonstrated that TNT formation participates in the pathogenesis of several diseases, including HIV^9–13^. However, the role of TNT or tumor microtubes in glioblastoma (GBM) have only recently been explored^14–23^.

Despite the aggressive treatment, the 2-year survival for GBM patients remains only in the 10–25% range, with few patients surviving beyond 5 years^24,25^. GBM treatment includes maximal surgical resection, if possible, followed by radiation therapy and the alkylator molecule temozolomide (TMZ)^26–30^. We and others demonstrated that TNT structures are induced in vitro and in vivo by TMZ and radiation treatment, suggesting that treatment alone could promote tumor adaptation or resistance in a TNT dependent manner^3,16,23^. Furthermore, our data demonstrated that TNT formation enables the spread of O^(6)^-Methylguanine-DNA methyltransferase (MGMT) between resistant and sensitive cells to TMZ and radiation treatment within the tumor to protect the tumor from treatment^23^. In addition to the MGMT transfer, we observed that mitochondria also transfer via TNT; however, whether mitochondria are truly transferred and functional in the recipient cell is unknown.

Mitochondria have many fundamental functions such as energy production, providing the precursors for multiple macromolecules such as lipids and nucleotides, and aiding differentiation, apoptosis, and the cell cycle^31–34^. In cancer, the association of mitochondrial dysfunction and carcinogenesis is well established^35–39^, but whether TNT formation and associated mitochondrial transfer participate in tumor adaptation to the microenvironment is unknown. Overall, malignant brain tumors show metabolic abnormalities as reflected by increased uptake of glucose^40^ and the use of unusual energy sources such as glutamate/glutamine that negatively correlate with GBM prognosis^41,42^. In tumor tissues, hypoxia occurs in specific tumor areas due to the inadequate blood vessel supply. Hypoxia induces tumor stem cell perpetuation and tumor resistance to chemotherapy^43,44^. Further, TNT has been demonstrated to accelerate mitochondrial exchange in multiple cell types^45,46^; however, whether mitochondrial transfer alters tumorigenesis is unknown. Here, we propose that TNT structures contribute to the exchange of tumor-derived mitochondria into neighboring healthy cells resulting in their adaptation to the new tumor-related metabolism and hypoxic conditions.

Our study demonstrated that TNT are formed between GBM cells and human primary astrocytes upon co-culture and stress conditions. We identified that mitochondria from tumor cells were transferred into primary astrocytes by a TNT-mediated mechanism. Transferred mitochondria were enlarged or fused and contained several genetic variations within the mitochondrial DNA (mtDNA), resulting in metabolic changes in the targeted cell to become more glucose- and glutamine-dependent. In addition, we observed that TNT communication between GBM cancer cells and astrocytes confers protection to non-tumor astrocytes from hypoxic conditions. Further, we identified TNT formation at the edge of the tumor using human primary resected GBM tumors suggesting that the mechanism proposed is present in vivo. Our findings demonstrate that TNT are critical mediators of cancer adaptation to the microenvironment and that blocking or reducing TNT communication can provide an alternative mechanism to therapeutically target GBM and other cancer types.

## Results

### A co-culture system to mimic the interface tumor-healthy tissue demonstrates that TNT formation is induced by oxidative stress

Several groups, including ours, have demonstrated that TNT formation is induced under several pathogenic conditions, including carcinogenesis^3,4,9,13,23,47–49^. In contrast, healthy adult cells express low to undetectable TNT levels^3,21^. Thus, during adulthood, TNT are detected mostly in pathogenic conditions.

**Figure 1:**
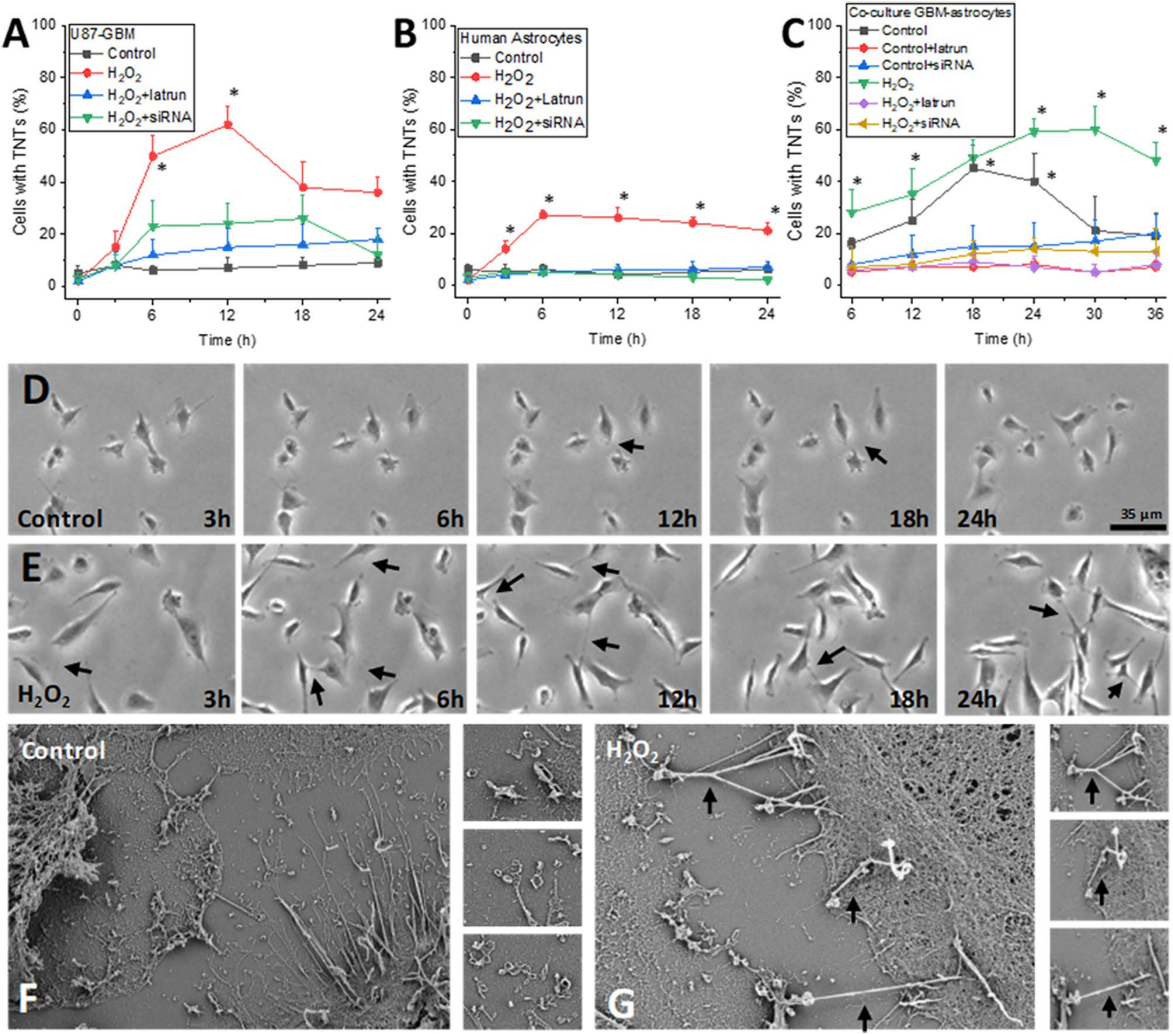
GBM cells communicate with human primary astrocytes via TNT. (**A**) Cultures of U87CD4CCR5 cells (U87GBM) in untreated (control) and H_2_O_2_ conditions indicate that oxidative stress increased the number of cells with TNT (*p ≤ 0.005 as compared to untreated control conditions, n = 6–8). Treatment with latrunculin (latrun) or siRNA to TTHY1(siRNA) prevented the formation of TNT induced by H_2_O_2_. (**B**) Quantification of TNT expressing human primary astrocytes (human astrocytes) in response to control and H_2_O_2_ conditions indicates a lower TNT formation than U87-GBM cells (*p ≤ 0.005 as compared to TNT blockers). Treatment with latrunculin (latrun) or siRNA to TTHY1(siRNA) prevented the formation of TNT induced by H_2_O_2_. (**C**) Time course of TNT formation between GBM cells, U87-GBM, and human primary astrocytes under control or H_2_O_2_ conditions. Treatment with latrunculin (latrun) or siRNA to TTHY1(siRNA) prevented the formation of TNT (*p ≤ 0.005 as compared to TNT blockers). (**D**) Representative time course of TNT formation in control conditions after 3, 6, 12, 18, and 24 h post imaging. (**E**) Time course of TNT formation upon H_2_O_2_ conditions, live-cell imaging after 3, 6, 12, 18, and 24 h. Arrows denote TNT structures. (**F**) Scanning electron microscopy of co-cultures of U87-GBM and primary human astrocytes under control conditions. Distinct filopodia are shown in the small inset images. (**G**) Denotes TNT induced by H_2_O_2_ conditions. Distinct TNT are shown in the small inset images, and at the bottom, it is possible to observe filopodia. Arrows denote TNT structures (cells analyzed per experiment were 35.3 ± 8.741, n = 6–8).

We used time-lapse microscopy to identify and quantify TNT formation by taking pictures every 0.5 or 1 min for 24 h. Here, we used two cell types: a well-characterized GBM cell line, U87MG cells transfected with CD4, and CCR5 (U87GBM), and human cortical primary astrocytes. Evaluation of TNT formation on pure cultures of U87-GBM cells (Fig. 1A) or primary human astrocytes (Fig. 1B) in basal conditions indicates low to undetectable levels of TNT formation (Fig. 1A,B, control). Oxidative stress induced by H_2_O_2_ treatment (100 µM) increased TNT formation transiently and aggressively, reaching up to ~ 60% of the GBM cells (U87CD4CCR5 or U87-GBM cells), showing TNT formation (Fig. 1A, H_2_O_2_, *p ≤ 0.005 as compared to untreated control conditions, n = 6–8). In contrast, treatment of human primary astrocytes with H_2_O_2_ resulted in a significant but modest increase in TNT formation as compared to U87-GBM cells (Fig. 1B, *p ≤ 0.005 as compared to untreated conditions, n = 8–12). The addition of TNF-α or LPS plus IFN-γ (100 ng/ml) did not further increase the number of TNT in primary astrocytes suggesting that cellular activation is not a condition that induces TNT formation in primary cells (data not shown). TNT formation induced by H_2_O_2_ was sensitive to TNT blockers such as low concentrations of Latrunculin (Latrun, 10 nM, disrupt microfilament organization by binding to monomeric g-actin) or siRNA to TTHY1 (Fig. 1A,B, siRNA, U87-GBM and human astrocytes, respectively). The efficiency of TTHY1 protein decrease for all the subsequent experiments reached 75.49 ± 17.09% of the control siRNA for all the experiments presented (n = 5 independent experiments). No alterations in filopodium formation, cell shape, or toxicity were observed with the TTHY1 siRNA or the control siRNA corresponding to the mouse pannexin-1 sequence (data not shown).

To model the interface between the tumor and the “healthy” surrounding cells, we generated a co-culture system between a well-characterized GBM cell type, U87-GBM, with human cortical primary astrocytes (Fig. 1C). The co-culture system consists of both cell types separated by a silicon barrier that, upon removal, allows both cell types to establish TNT between both cells, 45–50 µm. Using this system, we can directly evaluate TNT formation and transport between both cell types (Fig. 1C).

Upon removal of the silicon barrier in the co-culture model, TNT are formed from U87-GBM cells, into human primary astrocytes even without oxidative stress (Fig. 1C, control, *p ≤ 0.005 as compared to TNT blockers, n = 12–14). Overall, TNT formation in control conditions was mostly from U87-GBM cells into the human primary astrocytes (82.6 ± 5.69% of all TNT detected), indicating that tumor cells selectively targeted human primary astrocytes. H_2_O_2_ treatment further increased TNT formation between both cell types with an extended TNT formation time (Fig. 1C, *p ≤ 0.005 as compared to H_2_O_2_ plus TNT blocker conditions, n = 10–12). TNT formation between both cell types was sensitive to latrunculin (latrun, 10 nM at the time of barrier removal) or siRNA to TTHY1 (added 12–24 h before removing the barrier to the U87CD4CCR5 cells) treatment (Fig. 1C). A representative example of live cell imaging in control (Fig. 1D; 3, 6, 12, 18, and 24 h) and H_2_O_2_ conditions is showed (Fig. 1E; 3, 6, 12, 18, and 24 h, arrows represent the TNT structures). Scanning electron microscopy (SEM) of control cells (U87GBM and primary astrocytes) shows several filopodia without TNT (Fig. 1F, the small inserts show filopodia). Treatment with H_2_O_2_ induced the formation of TNT-like structures from the top optical plane of the cells (Fig. 1G, H_2_O_2_, arrows indicate TNT, and small insets show different TNT examples) that clearly illustrate the differences with filopodium attached to the substrate and no communicating neighboring cells. Overall, our data demonstrate that tumor GBM cells and primary astrocytes respond to oxidative stress by forming TNT; however, the function of TNT and associated communication remains unknown.

### Evaluation of TNT by transmission electron microscopy indicates that, in GBM cells, mitochondria at the TNT formation area are enlarged

To determine the biological differences between filopodium and TNT as well as the structures associated with TNT formation, we used transmission electron microscopy (TEM) to examine the top and bottom optical area of the cells using the coculture system described above. To perform these experiments, the bottom (filopodium localization) and the top (TNT localization) optical planes of the cells were sectioned and analyzed by TEM. We focused on the U87-GBM cells establishing TNT contacts with human primary astrocytes. Most of the TNT are generated from the U87-GBM cells into the primary astrocytes that function mostly as acceptors of the tumoral TNT.

**Figure 2:**
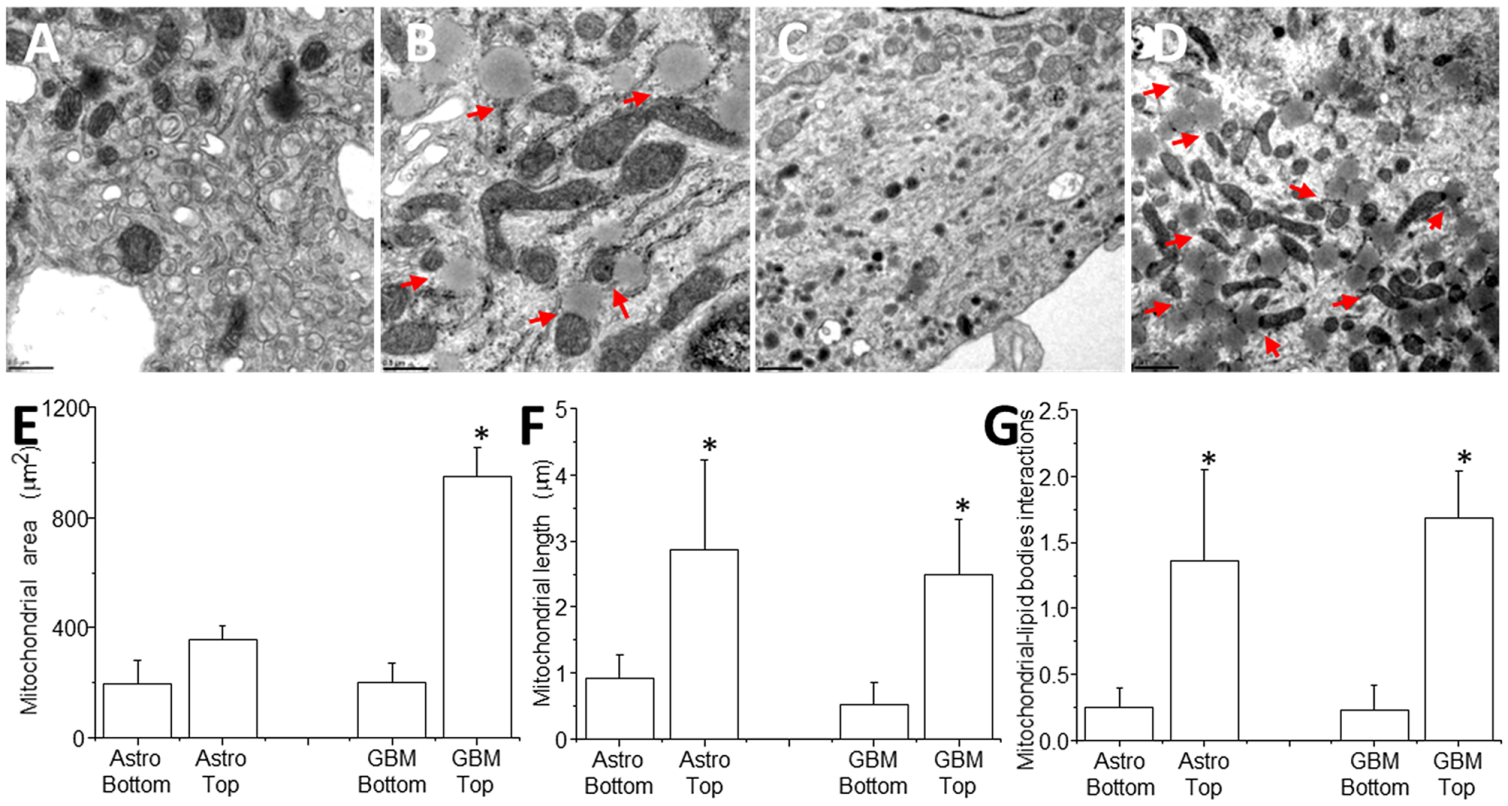
Mitochondrial heterogeneity in co-cultures of U87-GBM cells and human primary astrocytes. (**A**–**D**) Transmission electron microscopy (TEM) of co-cultures of GBM cells, U87-GBM, and human primary astrocytes indicates that tumor cells have a heterogeneous intracellular distribution of key organelles essential for TNT formation and transport. (**A**, **B**) Correspond to TEM from the same cells at the bottom and top optical sections of the cells, respectively. Arrows indicate the interactions between enlarged mitochondria and lipid bodies as well as the endoplasmic reticulum. Overall, top optical sections, where TNT are formed, present a bigger structure with more interactions with other organelles suggesting different metabolism. Arrows indicate these interactions. Bar: 0.5 µm. (**C**, **D**) Also, denote the same points of pictures (**A**) and (**B**), but at different magnification. Bar: 1 µm. (**E**) Quantification of the mitochondrial area at the top and bottom of cells in the co-culture system. GBM, top, has a higher mitochondrial area. (**F**) Quantification of mitochondrial length (µm) at the top and bottom sections. (**G**) Quantification of mitochondrial-lipid body interactions indicating the cells have intracellular heterogeneity and specific location associated with TNT formation (cells analyzed per experiment was 50 per condition, n = 5 independent experiment).

TEM analysis of U87-GBM in co-culture with human primary astrocytes cells indicates that in control conditions, the bottom section of the cells has normal size mitochondria with multiple vesicles (Fig. 2A,C, different magnifications, 0.5 and 1 µm, respectively). Analysis of top optical sections of the same cells, but now from the area where TNT are generated indicates a significant increase in mitochondrial size and strong interactions of mitochondria-lipid bodies with the endoplasmic reticulum, suggesting an intracellular heterogeneity that involves different organelle size, interactions, and potentially metabolism (Fig. 2B,D, see red arrows). These alterations in size and intracellular organelles represent a major discovery of cell heterogeneity and local metabolism as well as inter-organelle interactions (discussed in more detail later in the text). A similar mitochondrial dysfunction pattern and individual cell segregation were identified in human macrophages latently infected with HIV^50^. Differences in inter-organelle interactions have been observed in a growing number of pathologies and have been associated with alterations in mitochondrial dynamics, metabolic changes, and cell heterogeneity^51–55^; however, its role in cancer and TNT biology are unknown.

Quantification of the mitochondrial area at the top and bottom optical sections of both cell types in co-culture indicates a constant area used by mitochondria in astrocytes (Fig. 2E, Astro bottom, and top, n = 5 with 39–51 images analyzed). However, the analysis of the top and bottom of U87CD4CCR5-GBM cells in coculture with human primary astrocytes indicates that the bottom optical plane of the U87-GBM cells (GBM), close to the filopodium optical section, has a similar mitochondrial area to primary astrocytes in the same co-culture (Fig. 2E, GBM bottom as compared to astrocytes, top or bottom). In contrast, the mitochondria area at the top optical plane where TNT are generated (Fig. 2E, GBM top) was greater than the bottom area of the same cells analyzed. Thus, mitochondria are larger in the areas where TNT are formed, suggesting a metabolic specialization within the same cells that may contribute to TNT formation and associated transport.

Analysis of mitochondrial length indicates that mitochondria at the top optical level from U87-GBM cells, where TNT are formed, were larger than mitochondria at the bottom optical levels in both cell types in co-culture (Fig. 2F, *p ≤ 0.005 as compared to bottom pictures, n = 7). Interestingly, if we compare the data from Fig. 2E,F, mitochondria at the top optical section in astrocytes are bigger and equal in size than the mitochondria at the GBM top, suggesting that a TNT-mediated mechanism may share these enlarged mitochondria. Furthermore, at the top optical plane of the cells, a strong interaction of the enlarged mitochondria with lipid bodies and endoplasmic reticulum were observed, demonstrating a cellular and metabolic specialization or compartmentalization within the same cell in association with the area where TNTs are formed (Fig. 2G, *p ≤ 0.005 as compared to bottom pictures, n = 6–7).

### TNT formation between GBM cells and primary human astrocytes enables enlarged mitochondria to be transferred into human primary astrocytes

Several groups demonstrated that mitochondrial transfer via TNT could rescue apoptotic cells from apoptosis. The rescue process has been associated with the transfer of healthy mitochondria into compromised cells preventing their apoptosis^1,56–59^. However, whether the mitochondrial transfer via TNT and/or additional cargo modifies the metabolism of the targeted cell is unknown. In this section, several controls were included to detect any potential cross-contamination of our cocultures, including reversal media flow, amplification of CD4 transgene in the astrocytes (astrocytes are negative for CD4), and PTEN gene determination only present in U87 cells (see “Methods” for details). PCR for these genes was performed with no amplification up to 45 cycles using cocultures and laser captured material. As a positive control to evaluate contamination, U87CD4CCR5 cells (U87-GBM) were used. To identify the lower levels of PCR sensitivity for PTEN and CD4, we diluted 1–3, 10, 100 U87CD4CCR5 cells (positive for CD4 and PTEN) into 50,000 (K), 100 K, 500 K, and 1 million (M) primary astrocytes (negative for CD4 and PTEN). In all cases, positive detection of PTEN (C_T_, 1–3 U87CD4CCR5 cells into 50 k to 1 M primary astrocytes, 17.26 ± 6.54 cycles, n = 3 different cultures) and CD4 (C_T_, 1–5 U87CD4CCR5 into 50 k to 1 M primary astrocytes, 13.68 ± 8.57 cycles, n = 4 different cultures) genes were achieved suggesting that even 1–3 U87CD4CCR5 cells in a low–high number of primary astrocytes can be detected reliably. Higher numbers of U87CD4CCR5 cells diluted into primary astrocytes maintain or reduce the cycle numbers. Overall, our detection sensitivity was high and reliable for low levels of CD4 and PTEN. Overall, no contamination of these genes was detected in all the experiments presented in the current manuscript.

**Figure 3:**
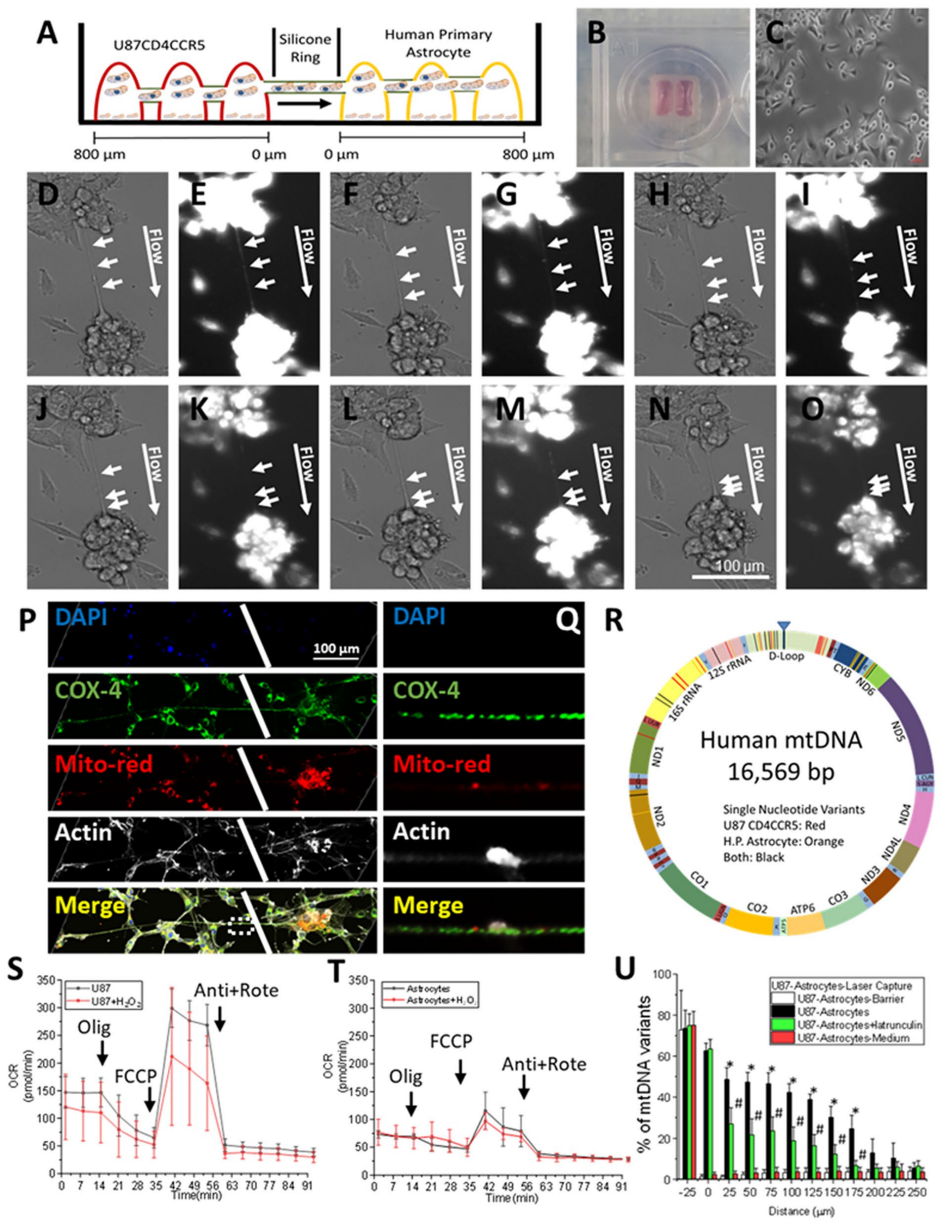
TNT transport “tumor” mitochondria from GBM cells into primary astrocytes. (**A**) To determine whether TNT mediate the transfer of mitochondria from GBM cells to primary astrocytes, we examined the formation of TNT through a co-culture system composed of GBM cells (U87CD4CCR5) and primary astrocytes. The co-culture model consists of both cell types separated by a silicon barrier. Upon removal of the barrier, the TNT formation is enabled. (**B**) A photograph of our co-culture model was previously described. (**C**) Image of the interface between our cell co-culture upon removal of the silicon barrier makes it possible to identify the separation between both cell lines. (**D**–**O**) The co-culture was stained with mitotracker orange, and the mitochondrial movement and cargo transfer were analyzed using time-lapse microscopy (**D**, **E**), 10 (**F**, **G**), 15 (**H**, **I**), 30 (**J**, **K**), 45- (**L**, **M**), and 60-min (**N**, **O**) post staining and after 12 h post removal of the silicon ring to enable TNT communication between U87CD4CCR5 cells and primary human astrocytes. (**P**, **Q**) To analyze the transfer of mitochondria from U87CD4CCR5 cells into primary astrocytes by TNT, the U87CD4CCR5 cells were stained with mitotracker orange, DAPI (nucleus), COX-4 (mitochondrial marker), and phalloidin (actin) upon removal of the silicon barrier. The formation of TNT with primary astrocytes is represented by the white line and represents the separation between U87CD4CCR5 cells and primary astrocytes. (**R**) Mitochondrial DNA sequencing (mtDNA) diagram, mtDNA indicates a specific fingerprint of single nucleotide variants in U87CD4CCR5 cells (red lines) and human primary astrocytes (orange lines). (**S**, **T**) Seahorse profile of U87CD4CCR5 and primary astrocytes in the presence and absence of H_2_O_2._ (**U**) Laser capture microdissection of the cell body and TNT was performed after co-culturing the cells on DIRECTOR laser microdissection slide in presence or absence of TNT blocker latrunculin (cells analyzed per experiment was 30 per condition).

First, we determined whether the enlarged mitochondria associated with TNT structures can be transferred from GBM cells into human primary astrocytes. We determined their movement using live-cell imaging (see schematic in Fig. 3A). Our co-cultures between U87CD4CCR5 cells and primary astrocytes were separated by a silicon ring, as shown in Fig. 3B, and live-cell microscopy was performed at the interface as shown in Fig. 3C. Co-cultures were stained with mitotracker orange, and mitochondrial movement and TNT-mediated transfer were quantified using time-lapse microscopy by taking still pictures every 0.5 or 1 min.

A representative time course example is presented in Fig. 3D–O, obtained after 5, 10, 15-, 30-, 45-, and 60-min post-staining and after 12 h post-removal of the silicon ring to enable TNT communication between U87CD4CCR5 cells and primary human astrocytes (Fig. 3D–O, arrows represent the mitochondria and their movement into primary astrocytes). Oxidative stress increased the numbers of mitochondria per TNT from 2.12 ± 1.43 in control conditions to 5.45 ± 3.21 in H_2_O_2_ treated conditions. Also, the mitochondrial speed inside TNT increased from 86.23 ± 16.38 to 143 ± 12.9 nm/s in H_2_O_2_ treated conditions as described for other tumors^46^.

A major observation is that mitochondrial transfer is unidirectional, from U87-GBM (U87CD4CCR5 cells) cells into primary astrocytes, and most of the mitochondria were enlarged, as observed in the SEM data. Second, TNT formation and the mitochondrial transfer were sensitive to latrunculin and siRNA to TTHY1 (see below), supporting an active TNT transport. To further examine the transfer of mitochondria from U87CD4CCR5 cells into primary astrocytes, we stained live U87CD4CCR5 cells with mitotracker orange, removed the silicon ring and allowed the formation of TNT with primary astrocytes, and then fixed and stained for DAPI (nucleus), COX-4 (mitochondrial marker), phalloidin (actin) as well as the mitotracker orange to examine mitochondrial transfer using confocal microscopy (Fig. 3P). Our data indicate that the signal for mitotracker orange (indicated in red) moved along TNT from U87GBM cells into primary astrocytes (Fig. 3P, the white line represents the separation between U87CD4CCR5 cells, U87-GBM, and primary astrocytes). We determined that TNT communication between both cell types reached distances of up to 500 µm; thus, the mitochondrial transfer can reach long distances from an area with tumor cells (Fig. 3Q). In addition, we determined whether the conditioned medium from U87CD4CCR5 cells could transfer mitochondria into the primary astrocytes to eliminate the possibility that extracellular vesicles such as exosomes could transfer the enlarged mitochondria into the primary astrocytes. Conditioned medium from U87CD4CCR5 cells containing exosomes was stained with mitotracker for 4–24 h and added to primary astrocytes for 24 h. Cells were fixed, and mitochondrial uptake was evaluated by confocal microscopy. We did not see any mitochondrial signal at any of the times tested. Overall, our data indicate that mitochondrial transfer between tumor and healthy cells was evident, unidirectional, and selective for enlarged mitochondria. Our next step was to determine whether the TNT-mediated mitochondrial transfer from tumor cells to “healthy cells” changes the metabolism of the targeted cell.

### mtDNA from tumor cells are shared with primary human astrocytes

To evaluate the metabolic effect of the TNT transferred mitochondria from U87CD4CCR5 cells (tumor) into primary astrocytes, we first determined whether tumor mitochondria were different than the mitochondria already present in primary astrocytes by DNA (mtDNA) sequencing and generating a SeaHorse profile to create a fingerprint of these organelles in each cell type. As indicated in Fig. 3R, mtDNA sequencing indicates a specific fingerprint of single nucleotide variants in U87CD4CCR5 cells (Fig. 3R, indicated in red lines) and human primary astrocytes (Fig. 3R, indicated in orange lines). The DNA mitochondrial variants present in both cell types compared to the Illumina database are indicated in black lines (Fig. 3R). Overall, we identified several single nucleotide variants (39), insertions (1), and deletions compared to the mtDNA variant analyzer v1.0.0. from Illumina. Our primary astrocytes set (n = 3) had variants in 73G, 146C, 152C, 263G, 296T, 315.1C, 514d, 515d, 750G, 921C, 1438G, 2045G, 2706G, 3107d, 4769G, 5046A, 14766T, 15110A, 15301A, 15326G, 15748C, 16124C, 16223T, and 16256T. In addition, the mtDNA sequencing of U87 cells (n = 3) indicates an overlapping but different profile than primary cells with 37 single nucleotide variants, five insertions, and one deletion. The specific variants are 73G, 146C, 195C, 263G, 315.1C, 513.1c, 513.2a, 513.3c, 513.4a, 750G, 1189C, 1438G, 1811G, 2706G, 3107d, 3480G, 4769G, 14766T, 14798C, 15326G, 16224C, 16278T, 16311C, 16390A, 16519C, and 16524G. Thus, each cell type has a unique mtDNA fingerprint that provides a unique signature for the mitochondria of each cell type.

To determine the metabolic profile of mitochondria in U87-GBM cells and primary astrocytes, we measured their oxygen consumption (OCR) using a SeaHorse analyzer (Agilent Technologies, Santa Clara, CA) to determine basal respiration, ATP production, proton leak, maximal respiration, and mitochondrial spare capacity (see details, https://www.agilent.com/en-us/products/cell-analysis-(seahorse)/mitochondrial-respiration-the-xf-cell-mito-stress-test). As indicated in Fig. 3S,T, U87CD4CCR5, and primary astrocytes, respectively, had a significantly different metabolism that was not affected by the application of H_2_O_2_ (Fig. 3S,T). Tumor cells have higher basal respiration, ATP production, proton leak, maximal respiration, and mitochondrial spare capacity than primary astrocytes (Fig. 3S,T), indicating that tumor cell metabolism in the conditions analyzed is more active than primary astrocytes. These data denote the metabolic differences between the tumor and primary cells as well as the potential to follow specific metabolic changes upon TNT formation and mitochondrial transfer.

### Laser capture microdissection indicates that tumor mitochondria diffuse into astrocytes in a significant manner

To further demonstrate that mitochondria from U87-GBM are transferred into primary astrocytes via TNT, we performed laser capture microdissection of cocultures of U87 (U87-GBM) and primary astrocyte cell bodies in the presence and absence of TNT blockers to identify whether GBM mtDNA could be identified in the primary astrocytes (Fig. 3U, 800 cell bodies per point).

Control samples using untreated and GBM-conditioned medium-treated pure cultures of primary astrocytes did not change the mtDNA profile of the primary astrocyte cells (Fig. 3U, astrocytes barrier). Removal of the silicon ring to enable TNT formation between GBM and primary cells resulted in increased TNT formation and mitochondrial transport, as indicated above. We determined that GBM mitochondria can reach distances up to 250 µm from the cellular interface. Laser captured material at different distances from the cellular interface (isolation of laser capture material every 25 µm, 0 µm represent the center of the silicon ring, and − 25 µm represent GBM cells) indicated that GBM mtDNA sequences diffused into primary astrocytes (Fig. 3U, black bars, U87-Astrocytes). The most commonly transferred sequences corresponded to the 513 variants (1c, 2a, 3c, and 4a), 16278T, 16311C, 16390A, 16519C, and 16524G when astrocyte cell bodies were analyzed (Fig. 3U). Blocking TNT formation with latrunculin (Latrun) or jasplakinolide (Jas, not shown) early after TNT formation in response to H_2_O_2_ treatment significantly prevented the mitochondrial sharing and diffusion of GBM mtDNA into primary astrocytes. This suggests that an active TNT transport is required for mitochondrial sharing into primary astrocytes (Fig. 3U, U87-Astrocytes + latrunculin, green bars). These data indicate that even though TNT can be formed between GBM and primary cells, an active transport mechanism is required to transfer mitochondria into the primary astrocytes. As a control, we used a conditioned medium from U87 cells (U87CD4CCR5, after 24, 48, and 72 h post culture) to evaluate whether exosomes containing mitochondria^60,61^ could transfer mtDNA into primary cells. No GBM mtDNA was detected in any of the laser captured fractions after exosomal exposure (Fig. 3U, U87-Astrocytes-medium, red bars). These data indicate that targeted delivery of mitochondria by a TNT-mediated mechanism is an active and directional process (Fig. 3U, − 25 µm).

### TNT formation and associated cargo transfer between GBM and primary cells, changing human primary astrocyte metabolism into a tumor-like metabolism

**Figure 4:**
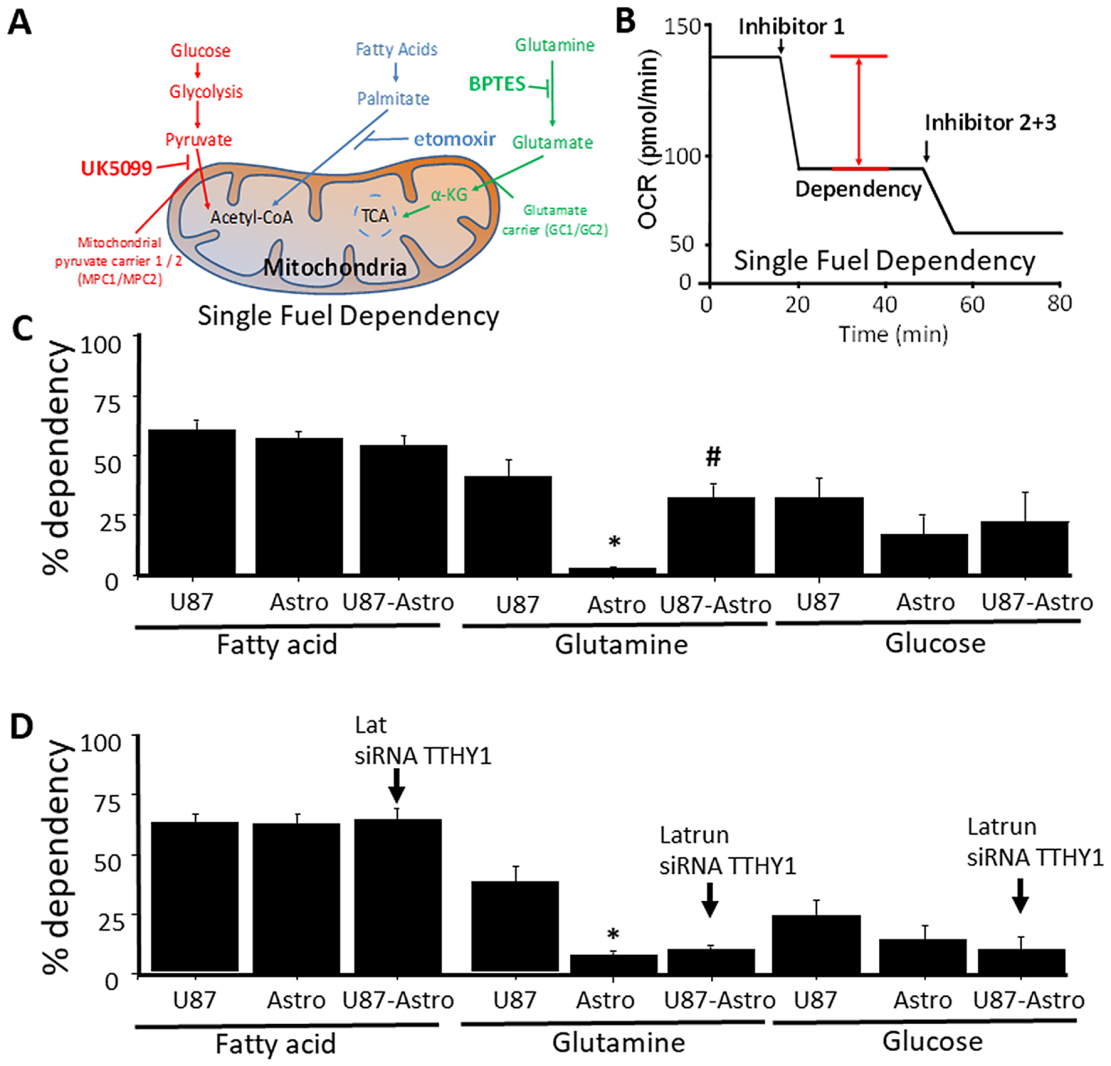
TNT between GBM and astrocytes enables the metabolic adaptation of primary astrocytes into a tumor-like metabolism. (**A**) Diagram of mitochondrial fuel input from glycolytic, glutamine, and fatty acid pathways, with appropriate inhibitors used to prevent usage of these pathways. (**B**) OCR changes are used to measure the percent dependency of one or two fuel types. OCR changes are measured at baseline (no compounds) for roughly 17 min, followed by injection of one fuel inhibitor for single fuel dependency or two inhibitors for double fuel dependency. OCR changes resulting from treatment are measured for approximately 40 min and followed by the injection cocktail. Single fuel dependency experiments were calculated as the percentage change from the baseline due to the first injection. (**C**) Mitochondrial OXPHOS dependency on fatty acid, glutamine, and glucose. U87-GBM cells (U87-GBM) were more dependent on glutamine than human primary astrocytes (*p = 0.0001 as compared to U87 cells, n = 4). There was no significant change in dependency on fatty acid or glucose for OXPHOS between GBM and primary astrocytes. However, upon co-cultures of U87-GBM cells with human primary astrocytes and formation of TNT as well as mitochondrial transfer, primary astrocytes began to use glutamine as a primary source of energy (U87-Astro, ^#^p ≤ 0.0002 as compared to pure cultures of astrocytes). No changes in fatty acid or glucose dependency were observed. No contamination was observed in the OXPHOS determinations with U87-GBM cells that expressed high levels of CD4, and astrocytes are CD4 negative. Also, no detection of deleted PTEN gene only present in U87 clones was detected. (**D**) Blocking TNT formation with latrunculin (Latrun) or siRNA for TTHY1 using a lentivirus prevented TNT formation, mitochondrial transfer, and the metabolic adaptation described above (cells analyzed per experiment was 41.43 ± 12.15, n = 4).

To determine whether the TNT transfer of GBM derived mitochondria could alter the metabolism of the targeted primary astrocytes, we determined the metabolic sources used for each cell type using single fuel dependency (https://www.agilent.com/cs/library/usermanuals/public/XF_Mito_Fuel_Flex_Test_Kit_User_Guide%20old.pdf). As described in Fig. 4A, we examined three different energy sources: glucose, fatty acids, and glutamine. In physiological conditions, most primary cells use glucose and fatty acids as a major energy source. However, in pathological conditions like neuro/glioblastoma and other types of cancer, amino acids such as glutamine are used as fuel sources^62^.

To measure fatty acid dependency, we measured OXPHOS by inhibiting fatty acid contribution to the TCA with etomoxir (fatty acid oxidation inhibitor), and later we applied two inhibitors together, a glucose oxidation inhibitor, UK5099, and a glutaminase inhibitor, BPTES, to examine the dependency on fatty acids as described in Fig. 4B. Pure cultures of GBM cells, U87-GBM (U87), mostly use fatty acids and glucose as a major energy source; however, at least half of the energy comes from glutamine (Fig. 4C, U87 glutamine, U87, and Astro, *p ≤ 0.004 as compared to U87 cells, n = 5). In contrast, human primary astrocytes obtain energy exclusively from fatty acids and glucose without using glutamine as a source of energy (Fig. 4C, Astro). Thus, the metabolism of each cell type is distinct.

To determine whether the cancer metabolic profile present in GBM cells, U87-GBM, could be transferred into human primary astrocytes via TNT, we determined the fuel dependency in astrocytes after coculture and TNT formation with U87CD4CCR5 cells (Fig. 4C, U87-Astro). U87 cells were eliminated from the culture by scraping (facilitated by the cell population's location). Analysis of human primary astrocytes in coculture with U87-GBM cells indicates that after 12–24 h of TNT communication, primary astrocytes change their metabolism to use glutamine to a similar extent as GBM cells (Fig. 4C, U87-Astro, ^#^p ≤ 0.0001 as compared to primary astrocytes without U87 TNT contact). No changes in glucose dependency were observed between the different cells and time points analyzed (Fig. 4C, glucose). In conclusion, TNT formation and mitochondrial transfer were associated with changes in human primary astrocyte metabolism, becoming tumor-like cells, especially for the dependency on glutamine as a major energy source.

To identify whether TNT formation mediates the adaptation of primary cells into a tumor-like metabolism profile, we repeated the experiments described above in the presence and absence of TNT blockers (Fig. 4D). Only recently has it been demonstrated in vivo that Tweety-Homolog 1 (TTHY1) protein is present in TNT or tumor microtubes and is a potent driver of tumor colonization and growth^16^. The use of two different TNT blockers, latrunculin (10 nM, latrun) or a lentivirus containing siRNA to TTHY1 (siRNA TTHY1, MOI, 0.1 M), did not affect fatty acid or glucose dependency of the astrocytes in co-culture with GBM cells (Fig. 4D, U87-Astro, see arrow). The use of a siRNA to mouse pannexin-1 as a negative control did not alter metabolic dependency (data not shown). However, the glutamine adaptation provided by GBM cells, U87, was not observed in the primary human astrocytes if TNT formation was prevented by latrunculin or the siRNA to TTHY1 (Fig. 4D, U87-Astro, siRNA TTHY1). Our data indicate that TNT formation is essential to transmit the metabolic adaptation observed in GBM cells into healthy primary astrocytes. The addition of conditioned medium from U87CD4CCR5 cells into human primary astrocytes at different time points, 12, 24, 48, and 72 h, did not replicate primary astrocyte adaptation to glutamine metabolism (data not shown). Also, we did not detect U87 PTEN-deleted expression in the astrocyte cultures, which validates the purity of our samples and the results presented.

### TNT formation and associated transport between GBM cells and primary human astrocytes protect primary cells from hypoxic conditions

Hypoxic conditions is a characteristic of glioblastoma tumors and neighboring tissues due to blood flow compromise, promoting a change in metabolism and survival of somatic and cancer stem cells that are believed to be responsible for tumor resistance and heterogeneity to chemotherapy and radiation^23^. Here, we examined whether TNT and their associated cargo between tumor cells, U87-GBM, and primary astrocytes can protect “healthy” cells from hypoxia toxicity.

**Figure 5:**
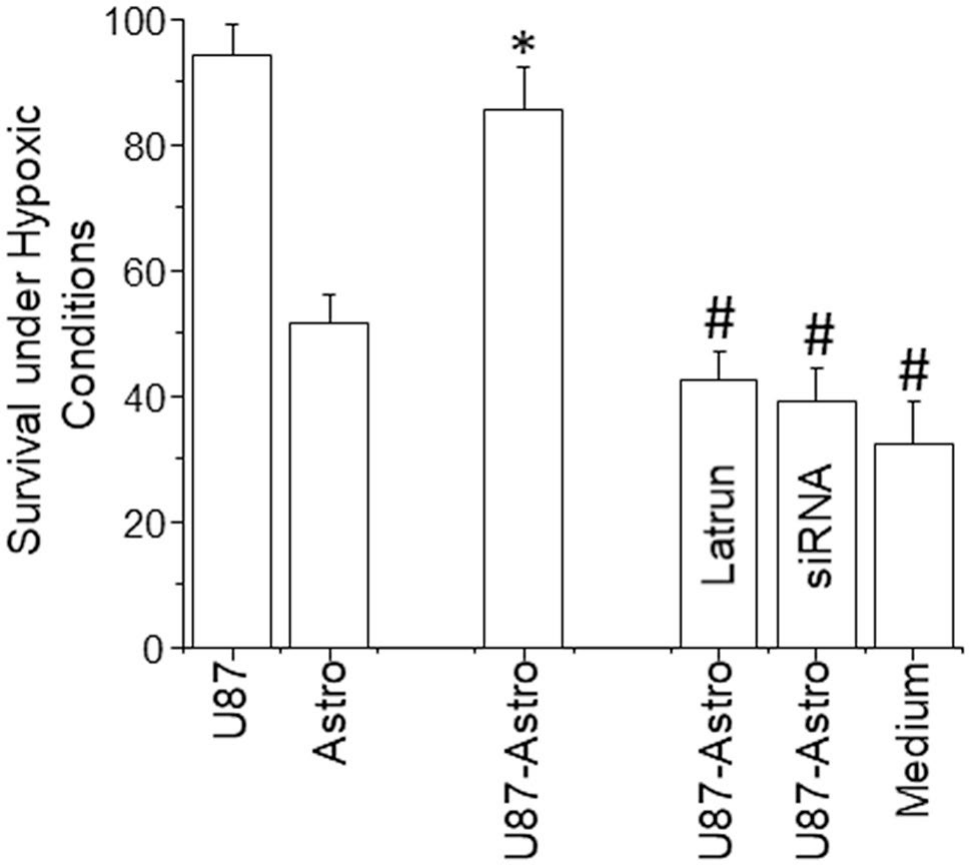
TNT-mediated transfer mediates adaptation to hypoxic conditions. A well-described adaptation of tumors to the microenvironment is the increased resistance to hypoxic conditions. Thus, to evaluate whether TNT mediates, in addition to the metabolic shift, adaptation to hypoxia for 72 h, survival of U87-GBM cells and primary cells was evaluated in the presence and absence of the TNT blockers, latrunculin (latrun), or siRNA to TTHY1 (siRNA). U87 cells (CD4CCR5), GBM, were insensitive to low O_2_ tension. Primary astrocytes were sensitive to low O_2_ tension conditions resulting in apoptosis (Astro). In contrast, the coculture of primary astrocytes with U87 cells and then quantification of apoptosis in the primary cells indicates that primary astrocytes upon TNT formation become adapted to hypoxic conditions (*p = 0.001 as compared to pure cultures of astrocytes, n = 7). Blocking the formation of TNT with latrunculin (latrun) or siRNA to TTHY1 (siRNA) prevented the adaptation of primary astrocytes to hypoxia. Also, using an siRNA to mouse pannexin-1 as a negative control did not alter TNT formation (data not shown). ^#^p ≤ 0.005 as compared to astrocytes present in the coculture and adapted to hypoxic conditions.

Using the co-culture model described above, we enabled GBM and primary astrocytes to form TNT for 24 h, and then hypoxic conditions were applied for 24, 48, and 72 h to quantify the survival of both cell types. U87-GBM cells were resistant to apoptosis when subjected to hypoxic conditions for 24, 48, and 72 h (Fig. 5, U87, only 72 h is shown). In contrast, human primary astrocytes were susceptible to hypoxic conditions (Fig. 5, Astro, only 72 h is shown). The co-culture of U87-GBM (U87) cells, which are resistant to hypoxic conditions, with human primary astrocytes, resulted in TNT formation and active transfer of mitochondrial and cytoplasmic material as described above. Quantification of astrocyte apoptosis within the co-culture system indicates that the formation of TNT with GBM cells, U87-GBM, resulted in a protective effect against hypoxia in the primary astrocytes (Fig. 5, U87-Astro, *p ≤ 0.005 as compared to pure cultures of astrocytes, Astro, n = 6). Thus, the human primary astrocytes then behaved like GBM tumor cells, surviving hypoxic conditions to protect the tumor and adapt the neighboring cells to tumor conditions. We identified that a minimum time of 12–24 h of TNT communication was required to transfer the GBM protective mechanism into human primary astrocytes. Blocking the formation of TNT with latrunculin (10 nM, latrun) or a lentivirus containing siRNA to TTHY1 (siRNA TTHY1, MOI, 0.1 M) prevented the adaptation of human primary astrocytes to hypoxic conditions (Fig. 5, U87-Astro + latrun or siRNA). Furthermore, isolation of small extracellular vesicles (exosomes as described^23^) or conditioned medium from U87 in basal or hypoxic conditions failed to protect human astrocytes against hypoxic conditions. This suggests that a soluble factor does not account for the protective phenotype (Fig. 5, Medium). Overall, our data indicate that TNT plays a key role in hypoxia adaptation to the tumor microenvironment.

### TNT formation at the interface tumor-“healthy” tissue is exacerbated

**Figure 6:**
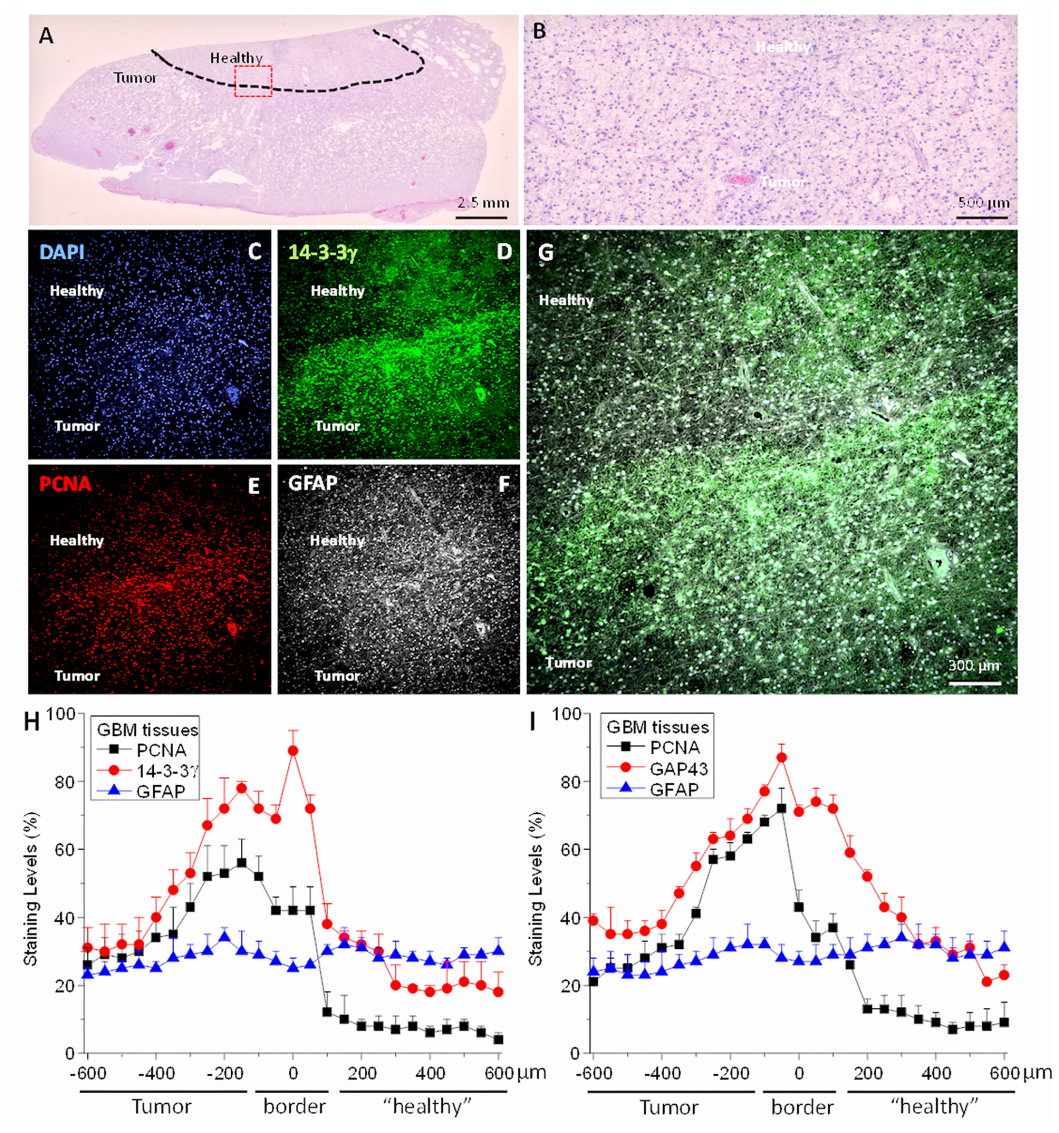
TNT are expressed in vivo at the interface tumor-healthy tissue. Primary GBM tumors were stained for H&E and immunofluorescence for DAPI (nuclear staining, blue staining), 14-3-3γ (TNT marker, green staining), PCNA (proliferation marker, red staining), GFAP (Glial marker, white staining), and merge colors. (**A**) Correspond to a large H&E of the tissue analyzed. (**B**) Amplification of the area denoted in red in the picture showed in A. The segmented line indicates the border of the tumor versus the healthy surrounding tissue. (**C**–**G**) Immune staining clearly indicates that protein 14-3-3γ (a TNT marker) is concentrated at the edge of the tumor, and most expression is from the tumor to the healthy surrounding areas (**D**). PCNA staining indicates preferential cellular proliferation in the tumor (**E**) and high glial activation at the tumor border as determined by GFAP staining (**F**). (**G**) Correspond to the merging of all the colors. (**H**) Correspond to the quantification of the fluorescence at different distances from the border or interface tumor-healthy tissue. Each point corresponds to the accumulated positive pixels every 40 µm into the tumor or healthy tissue for PCNA, 14-3-3-γ, and GFAP. (**I**) Correspond to the fluorescence quantification at different distances from the border or interface tumor-healthy tissue for PCNA, GAP43, and GFAP. In both graphs, the PCNA signal was significantly higher in the tumor (p = 0.0051 as compared to the healthy tissue. n = 4 different stage 4 GBM tumors from 4 different individuals). Proteins 14-3-3-γ and GAP43 were significantly concentrated at the tumor-healthy border, 400–300 µm (p = 0.00294 as compared to the healthy tissue. n = 4 different stage 4 GBM tumors from 4 different individuals). In contrast, GFAP in both graphs was high at the three areas analyzed, tumor, border, and healthy tissue (p = 0.143 as compared to the healthy tissue for all the points. n = 4 different stage 4 GBM tumors from 4 different individuals).

To determine whether TNT expression and localization occurs in vivo, we analyzed brain tissue sections from four different individuals with GBM. We focused on the interface between tumor and healthy tissue as described in our in vitro data. Brain tumor tissue sections were stained for H&E, DAPI (nuclear staining), protein 14-3-3γ (TNT marker), PCNA (proliferation marker), and GFAP (a glial marker) and analyzed by confocal microscopy with subsequent 3D reconstruction. As indicated in Fig. 6A, H&E staining denotes the tumor and a large portion of healthy tissue. Figure 6B corresponds to an amplification of the red square (interface) shown in Fig. 6A. All tumors analyzed had a significant proliferation (PCNA positive cells) and GFAP expression within the tumor and the tumor-tissue interface (Fig. 6E,F). Analysis of the protein 14-3-3γ and GAP43, both present in TNT and tumor microtubes^16,17,21,23^, were mainly concentrated at the interface between the tumor and the healthy brain tissue (Fig. 6D). Interestingly, protein 14-3-3γ and GAP43 were mainly localized in the cell body and the TNT-like processes at the interface between the tumor and the healthy brain area (Fig. 6G, green staining). We note the highly specific staining and TNT-like formation in all the tumors analyzed. Quantification of the distribution of the staining for TNT-associated proteins, 14-3-3γ (Fig. 6H) and GAP43 (Fig. 6I), as well as PCNA and GFAP, indicates the exquisite distribution of all these components at the tumor-tissue interface. Thus, TNT are present in vivo and mainly localized at the interface in human GBM samples.

## Discussion

The present study demonstrates first that (1) TNT mediate a long-range directed communication between GBM-GBM and GBM-primary astrocytes; (2) TNT are induced by oxidative stress; (3) TNT enable the transfer of enlarged mitochondria from cancer cells into neighboring primary astrocytes; (4) mitochondria from cancer cells are enlarged and have an altered metabolism that favors cancer development and tumor metabolism adaptation to the environment; (5) TNT generated in tumor cells protect surrounding non-tumor cells from hypoxia-induced apoptosis, probably to maintain tumor survival. Together, these results indicate the presence of a mechanism that drives TNT formation by tumor cells to target surrounding non-tumor cells to promote tumor growth and colonization. We propose that future studies in vivo could target TNT formation or associated transport to prevent tumor growth and treatment resistance.

It is currently accepted that there are several mechanisms of cross-talk between tumor and surrounding stromal cells, including soluble communication involving cytokines and exosomes, direct cell to cell contact-mediated by adhesion molecules, gap junction, and hemichannels, and the recently described TNT or tumor microtubes^17,21,23^. We show that TNT enables mitochondria exchange and other cytoplasmic factors from the tumor cell into the non-tumor cells. Mitochondrial exchange via TNT is not a new concept, as previously published by several groups^12,22,58,63^. The function of the mitochondrial transfer was suggested but not demonstrated. Here, we provide evidence that TNT-mediated transport of mitochondria changes the content and function of these organelles in the targeted non-tumor cell. The TNT-mediated mitochondrial transfer is based on an exquisite mechanism of intracellular organelle segregation within the same tumor cell and subsequent transfer of altered mitochondria into healthy cells. Inter-organelle interactions play a key role in health and disease conditions, including synaptic function, intracellular trafficking, lipid metabolism, and signaling^64,65^; however, the movement of these complexes within the cell or through TNT is unknown. We describe here that TNT-mediated transfer of mitochondria is a key event in non-tumor adaptation to tumor cells; however, our EM images indicate that mitochondria interact with the ER, Golgi, and lipid bodies. Thus, future studies will have to address the role of these organelles and their associated function in carcinogenesis mediated by TNT, including proliferation and metastasis. Only recently, the ER-mitochondrial microdomain association has been described as a “quasi-synapse” to enable Ca^2+^ transfer from the ER to the mitochondria, lipid synthesis and trafficking, cell death, bioenergetics, and autophagy, all processes altered in cancer^66^. We propose that TNT amplify these inter-organelle dysregulations into non-tumor cells to perpetuate carcinogenesis and adaptation/resistance to treatment.

Interestingly, we detected that TNT formation and associated mitochondrial transfer changes the metabolism and susceptibility to hypoxia of surrounding non-tumor cells. Even though we cannot quantify how many TNT-transferred mitochondria (there is an increase in 24.5 ± 8.09% of signal in the recipient cell from the GBM cells) are required to “flip” the metabolism of a non-tumor cell, at least 6 h of direct TNT communication were required to detect significant metabolic changes in the acceptor cell (primary astrocytes) by mtDNA sequencing and Sea Horse analysis. Overall, our data support a novel complex communication mechanism between the tumor and the surrounding tissue that could be targeted to reduce tumorigenesis.

More exciting is that TNT are minimally expressed under physiological conditions^2,21^; however, they proliferate upon stress or respond to several pathogens such as HIV and pathogenic conditions such as cancer^2,21,56^, Alzheimer’s^67^, Parkinson’s^57,68^, and other neurodegenerative diseases^69^. We hypothesize that TNT are mostly expressed in developmental conditions to coordinate targeted cell migration and differentiation, as nicely described during development^70–72^. However, these points open the possibility to target TNT to prevent or reverse disease therapeutically. According to our findings, several groups have suggested that TNT-transmitted organelles could alter the recipient cells' fate, but a clear readout has not been described. Some early examples have shown that among T lymphocytes, TNT can propagate cell death induced by Fas ligand^73,74^. Also, TNT have been proposed to play a key role in neurodegenerative diseases involved with the spread of aggregated proteins such as tau, APP, and Huntington by an intracellular pathway instead of a soluble mediated mechanism^9,67,75,76^. Other groups also propose that TNT can be used as a rescue mechanism to prevent long-term damage of compromised cardiomyocytes or endothelial cells by a direct TNT transfer of healthy mitochondria from mesenchymal stem cells^77,78^.

Our data indicate that cancer cells have at least two different kinds of mitochondria in the same cell suggesting cellular heterogeneity. Mitochondrial heterogeneity within a single cell has been correlated with chemotherapeutic resistance in mammalian cells^79,80^. While the mechanism is unknown, it is likely related to the multiple functions of this organelle, such as power source of the cell, apoptosis, calcium regulation, signaling, and free radical formation and control^81,82^. In our case, mitochondria close to filopodia were small as expected; however, in the same cell, mitochondria at the top of the cells, where TNT are formed, were larger and had strong interactions with lipid bodies suggesting alterations in metabolism and deficiency in mitochondrial fusion. Enlarged mitochondria have been associated with increased metabolism and stem cell survival under stress^83–85^, all conditions present in cancer. Our data indicate that most transferred mitochondria are enlarged, suggesting that TNT have a mechanism of mitochondrial selection.

The mitochondrial and lipid droplet interaction has been proposed as a general mechanism to rescue damaged cells and has been associated with several genetic diseases^86–89^. To denote the “power” of transferred mitochondria, microinjection of intact mitochondria into oocytes prevented apoptosis^90^. Mitochondrial transfer can also rescue aerobic respiration in compromised stem cells^91^ and recover epithelial function even if the mtDNA is damaged^92,93^. However, few studies demonstrate that TNT could be an efficient mechanism of mitochondrial transfer during disease conditions. Our data demonstrate that TNT generated between tumor and stromal cells promote the transfer of a unique type of mitochondria (enlarged and interacting with lipid bodies) into astrocytes resulting in a metabolic and hypoxic adaptation to the growing tumor. It is unknown whether the TNT-transferred mitochondria work independently or in concert with the host astrocyte mitochondria. Further, we can discard the possibility that additional soluble or organelle-associated factors transported via TNT into primary cells contribute to the metabolic and hypoxic adaptation. Our EM data indicate significant segregation of organelles within the areas where TNT are formed, including lipid bodies, ER, ribosomes, soluble enzymes, and lysosomes that can also contribute to tumor adaptation.

A critical “fingerprint” of the TNT-transferred mitochondria in U87 cells was their use of glutamine and glucose, and lipids to produce energy, in contrast to human primary astrocytes, which use only glucose and lipids. Glutamine use is a marker of aggressive GBM^42^. Glutamine is a nitrogen source used to synthesize nucleotides, amino acids, ATP, and a major anaplerotic precursor for the TCA cycle. Our data indicate that mitochondrial transfer enables “healthy cells,” such as primary astrocytes, to become tumor-like cells from a metabolic perspective. Glutamine dependency in different cancers promotes invasion and tumor aggressiveness^94^. Also, glutamine dependency can downregulate glycolysis reducing glucose uptake and lactate production, contributing to the Warburg effects observed in ischemic tumors^95,96^. In agreement, we identified that TNT formation also protects astrocytes from hypoxic conditions. Tumor persistence and growth rely on the survival of cancer stem-like cells promoted by hypoxic microenvironment^97–99^. It is believed that low oxygen levels prevent ROS formation and DNA damage, both nuclear and mitochondrial. Our data support both ideas that TNT-mediated mitochondrial transfer could change the metabolism of the targeted cell contributing to hypoxic condition adaptation—both essential components to promote tumor growth. Interestingly, TNT are the only communication system that enables targeted and selected pathogenic material transfer, including defective or compromised mitochondria. Thus, blocking TNT formation and associated communication could provide an additional treatment to reduce tumor adaptation and prevent chemotherapeutic resistance, as we recently demonstrated^86,100–104^. Also, as TNT are not expressed in healthy adult tissues, thus, we could expect minimal side effects.

We recently demonstrated TNT communication between heterogeneous GBM tumor cells based on their resistance to TMZ and radiation therapy^23^. First, surprisingly, we identified that anti-tumor treatment, TMZ, and radiation, promoted TNT formation and transport. Second, TNT concentrate the anti-apoptotic enzyme MGMT and distributes it among cells with insufficient MGMT expression to avoid apoptosis in response to TMZ and radiation treatment. Blocking TNT prevented MGMT diffusion and the survival of tumor cells with minimal MGMT expression. Third, we identified that tumor TNT are selective because MGMT protein, but not its mRNA, was transferred in vitro and in vivo in GBM and breast cancer^23^. In agreement, our data using human GBM tissues indicates that TNT are localized at the edge of the tumor, communicating tumor cells with the surrounding non-tumor cells to change their metabolism, probably to prime them to tumor development. These findings are outstanding and further support our data that TNT formation and associated transport are required for tumor metabolic adaptation^23^. Also, the localization of TNT at the edge of the tumor in vivo is remarkable, supporting the hypothesis that blocking TNT formation or associated communication may help prevent tumorigenesis and help kill cells or reduce tumor size.

## Methods

### Materials

RPMI, DMEM, fetal bovine serum (FBS), Penicillin/Streptomycin (P/S), and trypsin–EDTA were purchased from Thermofisher (Grand Island, NY). Phalloidin-conjugate to Texas red and anti-fade with DAPI were obtained from Thermo Fisher (Eugene, OR). Purified mouse IgG_2B_ and IgG_1_ myeloma protein were purchased from Cappel Pharmaceuticals, Inc. (Aurora, OH). DAPI, anti-rabbit, and anti-mouse conjugated to Alexa were from Thermo-Fisher (Eugene, OR). The in-situ cell death detection kit (TUNEL) was from Roche (Mannheim, Germany). siRNA was designed and synthesized by Origene. All protocols were evaluated and approved by UTMB.

### Glioblastoma cell lines

U87 MG cell line was purchased from the ATCC (Manassas, VA). U87 cells were transduced with CD4 and CCR5 (for coculture experiments, U87CD4CCR5 also named U87-GBM) grown in DMEM medium supplemented with 2–10% FBS, 1 µg/ml puromycin, 300 µg/ml G418, and pen/strep and maintained at 37 °C in a humidified incubator supplied with 5% CO_2_ as recommended by the HIV AIDS reagent repository. Three days before the co-culture, the antibiotics were removed to avoid any toxic effects in the primary cells. Mycoplasma test was performed every 4 months.

### Co-culture system

The co-culture model is comprised of U87CD4CCR5 cells (also named U87) and human primary astrocytes. Both cell types were initially separated by a silicon ring of a width of 50–150 µm; however, upon removal of the silicon ring, both cell types established TNT allowing the quantification of TNT at the interface as observed by live-cell imaging as we described^23^.

### Live cell imaging

Two different cell culture systems were used for analysis. Single-cell type cultures in regular tissue culture plates and the co-culture system described above. Confluence used was 50–70% to enable and record TNT extension and communication and reduce the possibility of overgrowth that can compromise TNT identification and characterization. Our imaging system comprises an Axio-observed Z1 with three redundant incubation systems with CO_2_ and humidity control to avoid any significant temperature, CO_2_, or humidity variations. Also, a media flow was used to prevent cell contamination between the co-cultures. We imaged for 24–48 h, recording every 30 s–1 min as we recently described^23^.

### TNT definition and quantification

Our criteria to identify TNT have been described in our previous publications^3,23,105^. They are based on; first, TNT are distinct from filopodia: in vitro, TNT are located on the top optical plane of the cell while filopodia are in the bottom optical plane of the cells. Thus, TNT are generated from a different cellular structure than filopodia. Currently, in primary human cells (neurons, astrocytes, macrophages, T cells, and microglia) or several cell lines, we do not have any evidence that filopodia become TNT; they are distinct structures^1,3,21,105,106^. Second, TNT do not attach to a substrate; instead, they are free-standing or in vivo associated with extracellular matrix components. Third, TNT communicate the cytoplasm of two or more cells at a minimal distance of 30 µm with open-ended and non-open-ended processes. Fourth, the TNT process can branch and reach distances up to 500 µm in vivo and in vitro^13,22,23,105,107^. Fifth, TNT can transport organelles, vesicular structures, and small molecules between TNT-connected cells. Sixth, TNT are positive for actin and negative or poorly positive for tubulin, a key difference as compared to filopodia, which is positive for both^1,89,108^. Seventh, TNT are positive for several TNT markers that do not present in filopodia, including TTHY1, GAP43, and protein 14-3-3γ^16^. Lastly, TNT and other similar structures defined by other groups^4,12,47,109^ corresponded to tangled processes with both characteristic, open-ended, and enclosed; however, whether other names such as cytonemes and tumor microtubes correspond to TNT needs to be determined. Thus, the definition of TNT structure is strict and separate from other cellular processes, including filopodium, lamellipodium, and pseudopods.

### Immunofluorescence

U87CD4CCR5 and human primary astrocytes were grown on glass coverslips, fixed, and permeabilized in 70% ethanol for 20 min at − 20 °C. Cells were incubated in a blocking solution for 30 min at room temperature and then in primary antibody overnight at 4 °C. Cells were washed several times with PBS at room temperature and incubated with phalloidin conjugated to Texas Red to identify actin filaments and/or the appropriate secondary antibody conjugated to FITC (Sigma, St. Louis, MO) for 1 h at room temperature, followed by another wash in PBS for 1 h. Coverslips were then mounted using an anti-fade reagent with DAPI, and cells were examined by confocal microscopy using an A1R Nikon confocal microscope with spectral detection (Tokyo, Japan) as we described^3,13,23^.

### Immunohistochemistry and analysis of human glioblastoma tumors

Postmortem brain tissue sections from GBM tumors (IV degree) were analyzed by Hematoxylin & Eosin (H&E) and four colors immunohistochemical staining for DAPI (nuclei staining) protein 14-3-3γ or GAP43 (both TNT markers), PCNA (a marker for proliferation), and GFAP (an astrocyte marker). Slices containing the tissue sections were blocked (5 mM EDTA, 1% fish gelatin, 1% essentially Ig-free BSA, 2% human serum, and 2% horse serum) for at least 60 min at room temperature and then incubated with anti-14-3-3γ or GAP43, PCNA, and GFAP overnight at 4 °C. The sections were washed with PBS, incubated with secondary antibodies for 1–4 h at room temperature, followed by serial washes in PBS for 1 h. Samples were then mounted using Prolong Gold anti-fade reagent and examined by confocal microscopy. Specificity was confirmed by replacing the primary antibody with the appropriate isotype-matched control reagent, anti-IgG_2A_, or the IgG fraction of normal rabbit serum (Santa Cruz Biotechnology) as we described^23^.

### Electron microscopy

Cells were fixed for 30 min at RT using 4% paraformaldehyde, 2% glutaraldehyde, buffered with 0.1 M sodium cacodylate. Cells were dried with hexamethyldisilazane until fully dry under a fume hood. The cells were analyzed using a Zeiss SUPRA 40 field emission scanning electron microscopy (SEM) and placed on a fitted mould for the holder. The holder was calibrated, and cells were imaged at various magnifications as indicated with an accelerating voltage of 3 kV. For transmission electron microscopy (TEM), a JEOL1200EX electron microscope was used. This protocol allowed the structure of the TNT, filopodia, and cell shape to be maintained. This protocol was recently described and optimized for TNT and associated structure preservation^23^.

### Mitochondrial DNA sequencing

For these experiments, a total of 100 ng of purified mitochondrial DNA was amplified using the Nextera XT DNA library prep kit (Illumina, Cat# FC-131-1024). In brief, using Illumina PCR primers (MTL-F1, MTL-R1, MTL-F2, MTL-R2), mitochondrial DNA from individual samples was amplified and evaluated on the Agilent BioAnalyzer DNA 12000 chip and a Qubit 3.0, using the dsDNA HS assay kit (Thermofisher, Cat# Q32854). The concentration and integrity of the samples were evaluated prior to subjecting them to tagmentation, a process by which the amplified mitochondrial DNA is fragmented and tagged with adapter sequences. Subsequently, the tagmented DNA of each sample was PCR amplified and indexed using different combinations of barcoded primers. The amplified DNA was cleaned up using AMPure XP magnetic beads (Beckman Coulter, Cat# A63881), and then libraries were normalized using Bioanalyzer-based normalization. In short, each PCR product was loaded onto a High Sensitivity DNA Chip, and each DNA sample library was normalized to 2 nM based on the molarity obtained from each electropherogram. Following normalization, samples were pooled, denatured, and diluted to yield a loading concentration of 6 pM. Sample libraries were then loaded onto the MiSeq v2 reagent cartridge and sequenced on a MiSeq instrument using a 2 × 251-cycle run with 2 index reads. Potential cell-to-cell contamination was assessed by evaluating the transgene's CD4 expression in the human primary astrocyte samples. Astrocytes are negative for CD4 expression^110–113^. None of the experiments presented CD4 transgene detected in the primary astrocytes discarding any cross-contamination of the samples. To ensure that mitochondrial transfer was not due to cell contamination, we determined the expression of the mutated gene PTEN in U87 cells^114^ in the primary astrocytes. Again, no contamination with this nuclear gene was found in the primary astrocyte fraction in any of the experiments performed.

### Hypoxia induction and assessment of astrocyte viability

U87CD4CCR5 and primary human astrocytes were cultured in a separate chamber or co-culture, as described above. Cell cultures were then placed in a polycarbonate hypoxia induction chamber (Billups-Rothenberg, Inc Del Mar, CA). A gas mixture containing 5% CO_2_ and 95% N_2_ was used for 10 min to purge the ambient air from the chamber and simulate an ischemic environment. To ensure the right temperature and humidity, the hypoxia chamber was sealed, humidified, and placed in a 37 °C incubator for 24–48 h. All experiments were repeated 6–8 times. Cell viability was assessed after 48 h by TUNEL staining and by quantifying the total numbers of cells remaining on the plate.

### Metabolic fuel flux or single fuel dependency assay

Metabolic Fuel Flux Assays were performed on XFe12 Bioanalyzer (Agilent). At 16–24 h post-TNT formation, all assays were performed following manufacture’s protocols and focused on the metabolism of primary astrocytes by eliminating the U87 cells from the plate. Briefly, the Mito Fuel Flex Test inhibits the import of three major metabolic substrates (pyruvate, fatty acids, and/or glutamine) with mitochondrial pyruvate carrier inhibitor UK5099 (2 mM), carnitine palmitoyltransferase 1A inhibitor etomoxir (4 mM), or glutaminase inhibitor BPTES (3 mM). This test determines cellular dependence on each of the metabolites to fuel mitochondrial metabolism by inhibiting the individual substrate import or the capacity for utilizing that substrate when the others are blocked. Baseline OCR was monitored for 18 min, followed by sequential inhibitor injections with OCR readings for 1 h following each treatment as described^50^.

### Image analysis

Raw data for TNT and other membrane protrusions were obtained using the Zen software (Zeiss Software, Germany). For confocal analysis, 3D deconvolutions were obtained using NIS elements (Nikon, Japan). Quantification of colocalization, intensities, lengths, and stability were performed in NIS elements and Image J.

### Statistical analysis

Information on the statistical tests used and the exact values of n (number of experiments) can be found in Figure Legends. All statistical analyses were performed using GraphPad Prism 6.0 (GraphPad Software Inc.) or origin PC software. The statistical tests were chosen according to the following: two-tailed paired or unpaired t-test was applied on datasets with a normal distribution (Kolmogorov–Smirnov test), whereas two-tailed Mann–Whitney (unpaired test) or Wilcoxon matched-paired signed-rank tests were used otherwise. p < 0.05 was considered as the level of statistical significance as described for other TNT manuscripts^3,13,23^.

### Ethical approval and informed consent

All protocols were carried out following the guidelines and regulations of the NIH and UTMB.

## Supplementary Information

Supplementary Information.
