# Supplementary material for: Tunneling nanotubes, TNT, communicate glioblastoma with surrounding non-tumor astrocytes to adapt them to hypoxic and metabolic tumor conditions
Source: Sci Rep. 2021 Jul 15;11:14556. doi: 10.1038/s41598-021-93775-8 (PMC8282675; doi:10.1038/s41598-021-93775-8)
Supplement: Supplementary file 1 — Supplementary Information. [file 41598_2021_93775_MOESM1_ESM.docx]

| REAGENT or RESOURCE | SOURCE | IDENTIFIER |
| --- | --- | --- |
| Antibodies | | |
| COX-4 Mouse mAb | Cell Signaling | Cat# 11967S; RRID:AB_2797784 |
| Goat anti-mouse IgG, AlexaFluor 647 | ThermoFisher Scientific | A21235; RRID:AB_2535804 |
| Donkey anti-rabbit IgG, AlexaFluor 488 | ThermoFisher Scientific | A21206; RRID:AB_2535792 |
| Biological Samples | | |
| U87 | ATCC | Cat# HTB-14 |
| Primary Astrocytes |  |  |
| Chemicals | | |
| DMEM | ThermoFisher Scientific | Cat# 11995-065 |
| H_2_O_2_ | Sigma-Aldrich | Cat# MKCL6061 |
| Latrunculin | Sigma-Aldrich | Cat# L5163 |
| Paraformaldehyde | Sigma-Aldrich | Cat# P6148 |
| Glutaraldehyde | Sigma-Aldrich | Cat# G5882 |
| Sodium Cacodylate | Sigma-Aldrich | Cat# C0250 |
| Fetal bovine serum | ThermoFisher Scientific | Cat# 16000044 |
| HEPES | ThermoFisher Scientific | Cat# 15630-080 |
| PenStrep | ThermoFisher Scientific | Cat#15140-122 |
| 0.05% Trypsin EDTA | ThermoFisher Scientific | Cat# 25300-054 |
| Ethanol | Sigma-Aldrich | Cat# E7023 |
| Fish gelatin | Sigma-Aldrich | Cat# G7041 |
| BSA (Bovine serum albumin) | Sigma-Aldrich | Cat# 05470 |
| Horse serum | Sigma-Aldrich | Cat# H0146 |
| Fluorescent Probes | | |
| ProLong™ Gold Antifade Mounting with DAPI | ThermoFisher Scientific | Cat# P36931 |
| MitoTracker Orange CMTMRos | ThermoFisher  Scientific | Cat# M7510 |
| F-Actin (Phalloidin 680) | ThermoFisher  Scientific | Cat# A22286 |
| Software | | |
| NIS-Elements-AR | Nikon | [https://www.microscope.healthcare.nikon.com](about:blank) |
